# The Impact of a Nitric Oxide Synthase Inhibitor (L-NAME) on Ischemia–Reperfusion Injury of Cholestatic Livers by Pringle Maneuver and Liver Resection after Bile Duct Ligation in Rats

**DOI:** 10.3390/ijms20092114

**Published:** 2019-04-29

**Authors:** Junji Iwasaki, Mamdouh Afify, Christian Bleilevens, Uwe Klinge, Ralf Weiskirchen, Julia Steitz, Michael Vogt, Shintaro Yagi, Kazuyuki Nagai, Shinji Uemoto, Rene H. Tolba

**Affiliations:** 1 Institute for Laboratory Animal Science and Experimental Surgery, RWTH-Aachen University, Medical Faculty, 52074 Aachen, Germany; junji.i@kuhp.kyoto-u.ac.jp (J.I.); mamdouh.afify@gmail.com (M.A.); jsteitz@ukaachen.de (J.S.); mvogt@ukaachen.de (M.V.); 2 Two Photon Imaging Facility of the Interdisciplinary Center for Clinical Research (IZKF), RWTH-Aachen University, Medical Faculty, 52074 Aachen, Germany; 3 Department of Pathology, Faculty of Veterinary Medicine, Cairo University, Giza Square 12211, Egypt; 4 Department of Anesthesiology, RWTH-Aachen University, Medical Faculty, 52074 Aachen, Germany; cbleilevens@ukaachen.de; 5 Department of General, Visceral and Transplantation Surgery, RWTH-Aachen University, Medical Faculty, 52074 Aachen, Germany; uklinge@ukaachen.de; 6 Institute of Molecular Pathobiochemistry, Experimental Gene Therapy and Clinical Chemistry, RWTH-Aachen University, Medical Faculty, 52074 Aachen, Germany; rweiskirchen@ukaachen.de; 7 Division of Hepatobiliary Pancreatic and Transplant Surgery, Graduate School of Medicine, Kyoto University, Kyoto 606-8501, Japan; shintaro@kuhp.kyoto-u.ac.jp (S.Y.); kaznagai@kuhp.kyoto-u.ac.jp (K.N.); uemoto@kuhp.kyoto-u.ac.jp (S.U.)

**Keywords:** bile duct ligation, ischemia–reperfusion injury, liver, nitric oxide synthase, Pringle maneuver

## Abstract

The Pringle maneuver (PM) has been widely used to control blood loss during liver resection. However, hepatic inflow occlusion can also result in hepatic ischemia–reperfusion injury (IRI), especially in patients with a cholestatic, fibrotic, or cirrhotic liver. Here we investigate a nitric oxide synthase (NOS) inhibitor N-Nitroarginine methyl ester (L-NAME) on IRI after the PM and partial hepatectomy of cholestatic livers induced by bile duct ligation (BDL) in rats. Control group (non-BDL/no treatment), BDL + T group (BDL/L-NAME treatment) and BDL group (BDL/no treatment) were analyzed. Cholestasis was induced by BDL in the L-NAME and BDL group and a 50% partial hepatectomy with PM was performed. L-NAME was injected before PM in the BDL + T group. Hepatocellular damage, portal venous flow, microcirculation, endothelial lining, and eNOS, iNOS, interleukin (IL)-6, and transforming growth factor-β (TGF-β) were evaluated. Microcirculation of the liver in the BDL + T group tended to be higher. Liver damage and apoptotic index were significantly lower and Ki-67 labeling index was higher in the BDL + T group while iNOS and TGF-β expression was decreased. This was corroborated by a better preserved endothelial lining. L-NAME attenuated IRI following PM and improved proliferation/regeneration of cholestatic livers. These positive effects were considered as the result of improved hepatic microcirculation, prevention of iNOS formation, and TGF-β mRNA upregulation.

## 1. Introduction

Excessive blood loss from the raw surface during transection of the liver parenchyma is associated with a poor postoperative outcome, which can lead to liver failure, especially when the remnant liver is small [1]. The Pringle maneuver (PM) is a continuous or intermittent portal triad clamping technique for nonselective inflow occlusion that has been used widely to control blood loss during liver resection [2,3,4]. However, hepatic inflow occlusion can also result in hepatic ischemia–reperfusion injury (IRI) [5,6], especially in patients with a cholestatic, fibrotic, or cirrhotic liver [7,8,9].

Nitric oxide (NO) is a radical gaseous inflammatory mediator that is produced from the amino acid L-arginine through a reaction catalyzed by the NO synthase (NOS) isoforms. NO plays various roles throughout the body, yet whether or when NO has cytotoxic or cytoprotective effects is controversial. There are three main isoforms of NOS, including neuronal NOS (nNOS), inducible NOS (iNOS), and endothelial NOS (eNOS). nNOS and eNOS are constitutively expressed NOS (cNOS) isoforms and produce relatively small amounts of NO. In contrast, iNOS is upregulated in the liver under various conditions such as endotoxemia, hemorrhagic shock, ischemia–reperfusion, hepatitis, and liver regeneration. This isoform synthesizes NO at high concentrations for extended periods of time. iNOS is expressed in different types of cells, including endothelial cells, hepatocytes, Kupffer cells, and smooth muscle cells. N(ω)-nitro-L-arginine methyl ester (L-NAME) is an L-arginine analog and a nonselective NOS inhibitor that strongly inhibits eNOS as well as iNOS through competitive inhibition.

It is known that NO production in a tissue is continual, keeping balance between vasoconstriction and vasodilatation [10]. In this context, NO and its derivatives are known to play important roles in the pathophysiology of the liver [11]. In general, it is assumed that NO derived from endothelial NO synthetase (eNOS) in liver sinusoidal endothelial cells is protective against disease progression, while inducible NOS (iNOS)-derived NO contributes to pathological processes by acting as a pro-inflammatory mediator [11]. Therefore, strategies that target deleterious NO effects are intensively discussed as potential new therapies and experimentally investigated in many disease models.

In this study, we investigated the therapeutic effect of L-NAME on IRI following PM in cholestatic remnant livers. Therefore, cholestatic livers were induced by ligation of the common bile duct (BDL), which has been widely used to study cholestatic liver injury; cholestasis is known to occur 14 days after BDL [12,13,14,15].

## 2. Results

**Figure 1:**
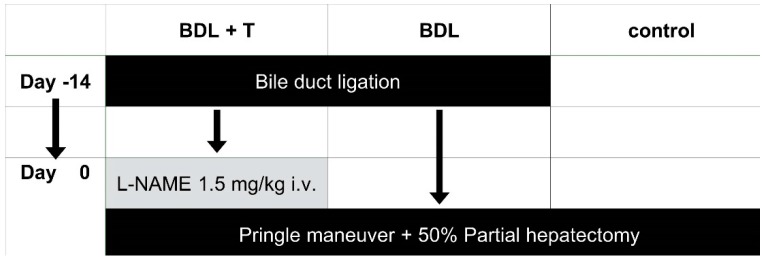
To investigate the effect of a nitric oxide synthase (NOS) inhibitor on ischemia–reperfusion injury (IRI) after the Pringle maneuver and partial hepatectomy of cholestatic livers induced by bile duct ligation (BDL) rats underwent BDL surgery to induce biliary cirrhosis in the BDL + T and BDL groups. Fourteen days later, 50% partial hepatectomy with PM was performed in all groups. The rats in the BDL + T group were additionally intravenously injected with N-Nitroarginine methyl ester (L-NAME) (1.5 mg/kg) 15 min before PM.

The aim of this study was to investigate the effect of a NOS inhibitor on IRI after the Pringle maneuver and partial hepatectomy of cholestatic livers induced by bile duct ligation (BDL) in rats. We analyzed three groups, (BDL+ T, BDL, and control) according to the time line depicted in Figure 1.

### 2.1. Hepatocellular Damage

**Figure 2:**
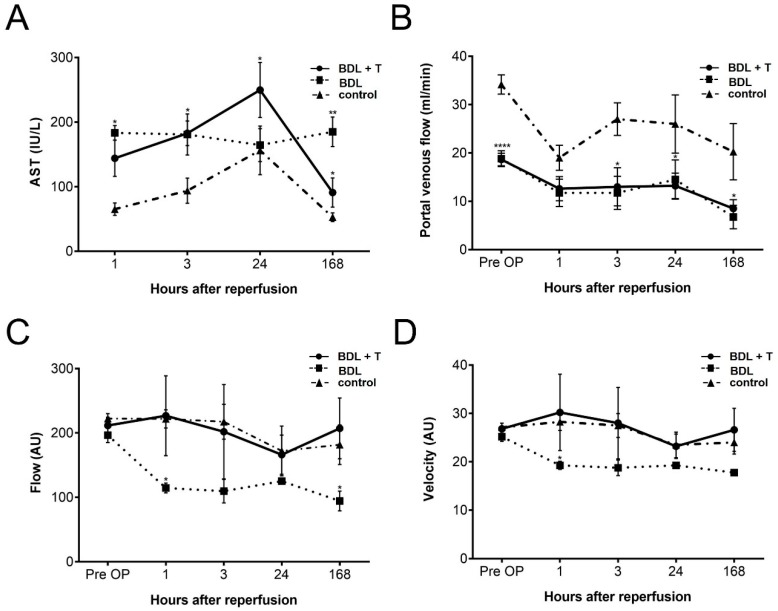
Hepatocellular damage, circulation, and microcirculation in the different treatment groups were analyzed based on (**A**) serum aspartate aminotransferase (AST) levels, (**B**) portal venous flow, (**C**) flow, and (**D**) velocity pre-operative, 1, 3, 24, and 168 h after reperfusion. Mean and standard deviation are shown in each group with significance levels of * *p* < 0.05, ** *p* < 0.01, **** *p* < 0.0001.

Following standard procedures, hepatocellular damage was evaluated by measuring serum levels of aspartate aminotransferase (AST) at different time points (Figure 2A). The BDL + T group showed lower AST levels than that of the BDL group 1 h after reperfusion, but showed the highest levels among the three groups after 24 h of reperfusion. At 168 h after reperfusion, AST levels of the BDL group were significantly higher than those of the BDL + T and control group. There was no significant difference in AST levels between the BDL + T and BDL group at other time points. AST levels of the control group were significantly lower in comparison to the BDL + T (168 h) and BDL (1 h, 3 h, 24 h, 168 h) treatment group. Cholestasis makes a chronic injury, in which the mechanical blockage in the duct system prevents the bile flow from the liver to the duodenum. This provokes a strong accumulation of bile in the hepatic parenchyma resulting in a significant increase in AST.

### 2.2. Portal Venous Flow (PVF)

PVF was measured before PM and at each time point of sacrifice. The PVF of rats in the BDL + T and BDL group was lower than in the control group (Figure 2B). There was no significant difference between the BDL + T and BDL group. The control group showed the highest PVF at any time point after reperfusion among the three groups, and there were significant differences before PM and 3, 24, and 168 h after reperfusion (*p* < 0.05).

### 2.3. Microcirculation of the Liver

The microcirculation of the liver was evaluated by laser Doppler flowmetry before PM and at each time point of sacrifice (Figure 2C,D). Although flow and velocity of the BDL group decreased gradually after reperfusion, the BDL + T and control group maintained microcirculation of the liver for 168 h after reperfusion. However, there were no significant differences among the groups at any time points of sacrifice except at 1 h after reperfusion between the BDL + T and BDL group.

### 2.4. Lipid Peroxidation

To estimate the oxygen free radical activity in the liver, we evaluated lipid peroxidation in serum by measuring malondialdehyde (MDA) levels at 1, 3, 24, and 168 h after reperfusion (Figure S1). The MDA levels were lower in the control group compared to the two other groups. Significant differences were only seen between the BDL + T and the control group at 1 h and 3 h after reperfusion.

### 2.5. Pro-Inflammatory Cytokines

**Figure 3:**
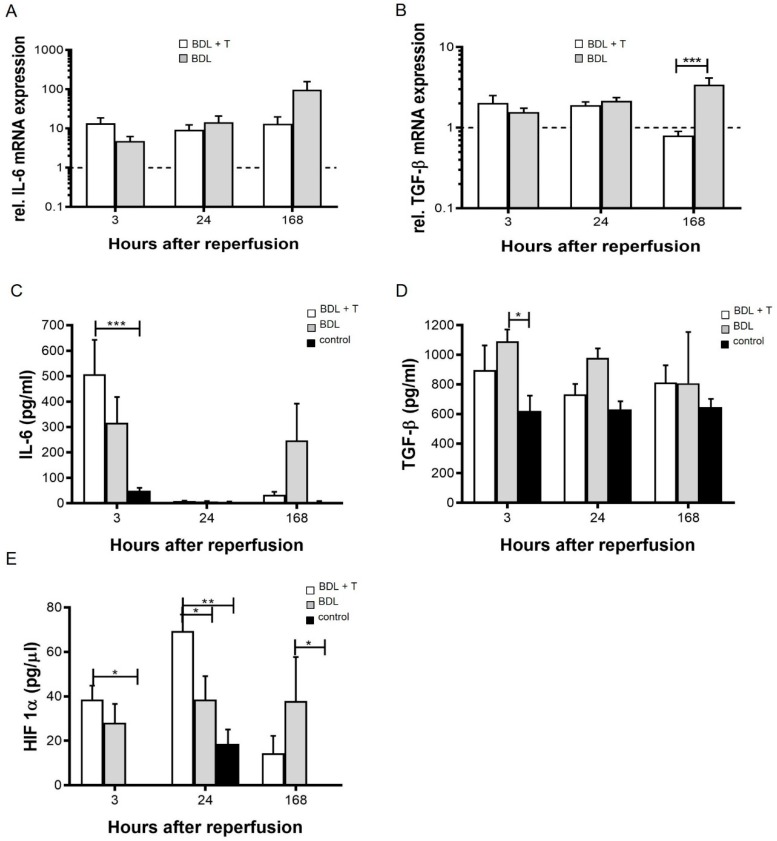
As an important feature of tissue fibrosis and epithelial–to–mesenchymal transition (EMT) pro-inflammatory cytokines (**A**,**B**) interleukin (IL)-6, (**C**,**D**) transforming growth factor-β (TGF-β) mRNA, and protein levels and the (**E**) hypoxia-inducible factor-1α (HIF-1α) levels were analyzed in serum 3, 24, and 168 h after reperfusion. Mean and standard deviation are shown in each group with significance levels of * *p* < 0.05, ** *p* < 0.01, *** *p* < 0.001.

Treatment induced changes in interleukin (IL)-6 and TGF-β expression were measured on mRNA and protein levels (Figure 3A–D). The expression of IL-6 mRNA tended to be higher in the BDL group than in the BDL + T group at 168 h after reperfusion, but the difference was not significant (*p* = 0.09; Figure 3A). Relative TGF-β1 mRNA expression at 3 and 24 h after reperfusion was comparable in the BDL + T and the BDL group. However, its expression at 168 h after reperfusion was significantly higher in the BDL group than in the BDL + T group (*p* < 0.0004; Figure 3B). Serum IL-6 and TGF-β levels were measured by ELISA at 3, 24, and 168 h after reperfusion to evaluate the inflammatory response caused by ischemia–reperfusion (Figure 3C,D). Serum IL-6 levels in the BDL + T group already decreased 24 h after reperfusion, and the BDL group showed high, but not significant, different IL-6 levels at 3 h and 168 h after reperfusion (3 h: 316.9 ± 101.0 pg/mL, 168 h: 247.45 ± 144.3 pg/mL). Significantly higher levels of IL-6 in the BDL + T group was observed only in comparison to the control group at 3 h after reperfusion (BDL + T group, 507.4 ± 135,1 pg/mL; control group, 50.2 ± 10.72 pg/mL; *p* < 0.001; Figure 3C).

Transforming growth factor-β (TGF-β) serum levels in the BDL group tended to be higher than that of the other two groups (Figure 3D). However, there were no significant differences among the three groups at each time point after reperfusion except for time point at 3 h, here TGF-β serum levels in the BDL group was significantly higher than in the control group. In summary, the control group showed the lowest values of IL-6 and TGF-β throughout the observation period.

### 2.6. Serum HIF-1α Levels

Serum HIF-1α levels were measured after 3, 24, and 168 h of reperfusion (Figure 3E). HIF-1α levels at 3 h after reperfusion were comparable between the BDL + T and BDL group. The BDL + T group showed the highest levels among the three groups at 24 h after reperfusion (69.6 ± 7.4 pg/µL), and there was a significant difference compared with the BDL group (38.6 ± 10.5 pg/µL; *p* < 0.05). The HIF-1α level in the BDL group at 168 h after reperfusion tended to be higher than that of the BDL + T group, but no significant difference was observed. Animals in the control group showed in general significant lower levels of HIF-1α in comparison to the BDL and/or BDL + T group (3 h, BDL + T vs. control, *p* < 0.05; 24 h, BDL + T vs. control, *p* < 0.01; BDL vs. control, *p* < 0.05; 168 h, BDL vs. control, *p* < 0.05).

### 2.7. NOx Levels in Liver Tissue

**Figure 4:**
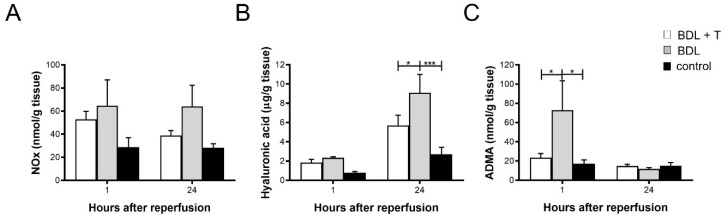
To measure the intensity of liver damage (**A**) NOx levels, (**B**) Hyaluronic acid (HA), and (**C**) Asymmetric dimethylarginine (ADMA) levels in liver tissues were evaluated 1 h and 24 h after reperfusion. Mean and standard deviation are shown in each group with significance levels of * *p* < 0.05 and *** *p* < 0.001.

Hepatic NOx levels were measured after 1 h and 24 h of reperfusion (Figure 4A). The control groups showed the lowest levels among the three groups, whereas the BDL group showed higher but not significantly higher levels of NOx than the animals in the BDL + T group.

### 2.8. Hepatic Hyaluronic Acid (HA) Levels

HA in liver tissue was measured as parameter for endothelial cell function at 1 and 24 h after reperfusion (Figure 4B). There was no difference among the three groups at 1 h after reperfusion. However, at 24 h after reperfusion, the HA level of the BDL + T group (5.70 ± 1.06 µg/g) was significantly lower than that of the BDL group (9.09 ± 1.91 µg/g; *p* < 0.05). The BDL group showed significantly higher HA levels in comparison to the control group (*p* < 0.001).

### 2.9. Asymmetric dimethylarginine (ADMA) in Liver Tissue

ADMA in liver tissue was measured at 1 h and 24 h after reperfusion (Figure 4C). The ADMA level in the BDL group at 1 h after reperfusion (72.3 ± 30.6 nmol/g) was significantly higher than that of the BDL + T group (23.3 ± 4.57 nmol/g, *p* < 0.05) and of the control group (17.2. ± 3.9 nmol/g, *p* < 0.05). There was no difference in the ADMA level among the three groups at 24 h after reperfusion.

### 2.10. Expression of iNOS and eNOS

**Figure 5:**
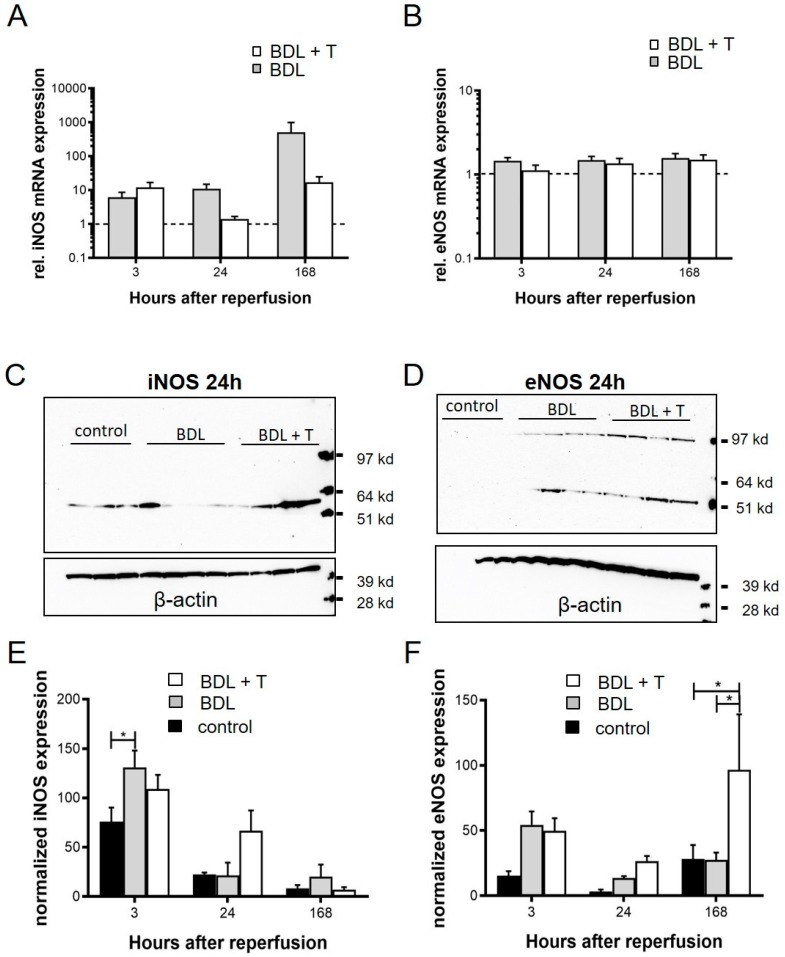
For the analysis of the effect of the L-NAME treatment (BDL + T) inducible nitric oxide synthase (iNOS) and endothelial nitric oxide synthase (eNOS) mRNA levels (**A**,**B**) and protein expression (**C**,**D**) were determined and quantified by densitometry (**E**,**F**). Relative mRNA expression of iNOS (A) and eNOS (B) were measured by RT-qPCR after 3, 24, and 168 h after reperfusion (C, D). Results of protein expression in Western blots are shown 24 h after reperfusion. Densitometry analysis of Western blot results of iNOS (E) and eNOS (F) protein expression. β-actin normalized expression of iNOS and eNOS after 3, 24, and 168 h after reperfusion are shown. Mean and standard deviation are shown in each group with significance levels of * *p* < 0.05.

The expression of iNOS and eNOS was analyzed on the level of relative mRNA expression using quantitative real-time PCR (RT-qPCR; Figure 5A,B) as well as on the level of protein expression using Western blot (Figure 5C,D) techniques. In the BDL group, relative iNOS mRNA expression was increased at the 168 h time point compared with that at 3 h, this elevation was not observed in the BDL + T group (Figure 5A). In contrast, relative eNOS mRNA expression was comparable between the BDL + T and BDL groups at each time point investigated (Figure 5B). In contrast to the iNOS mRNA result the iNOS protein expression by Western blot showed a higher protein content in the BDL group compared to the BDL + T group visible in the blot itself (Figure 5C) and as analyzed by densitometry of the Western blots. Significant differences were only seen between the BDL group and the control group at 3 h after reperfusion. The eNOS Western blot results were absolutely comparable to the eNOS mRNA expression and showed no difference between the groups at 24 h after reperfusion as shown in the Western blots and after densitometry analysis of the blots. However, significant differences could be detected with densitometric analysis of the liver samples of the BDL + T group compared to the BDL and control group (*p* < 0.05). In addition, proteolytic degradation of eNOS may play a role in post-ischemic endothelial dysfunction. Loss of eNOS activity after ischemia has been shown to result from a combination of intracellular acidosis-dependent protein denaturation leading to proteolytic degradation [16].

### 2.11. Histopathology and Immunohistochemistry

**Figure 6:**
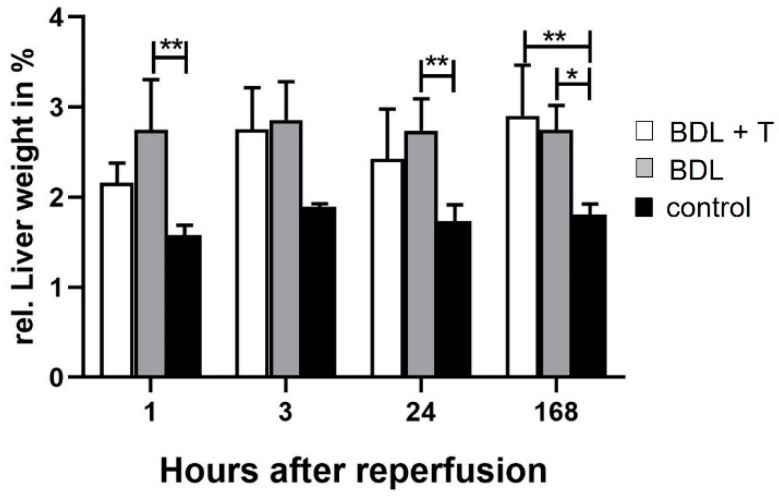
Changes at relative liver weight (in %) were evaluated in animals after 1, 3, 24, and 168 h after reperfusion in the BDL + T, BDL, and control group. Mean and standard deviation are shown in each group with significance levels of * *p* < 0.05 and ** *p* < 0.01.

For pathological and histopathological analysis, the weight of the livers of animals from the BDL + T, BDL, and control group was measured and relative organ weights were calculated. No significant differences could be observed at the relative liver weight between the BDL + T and the BDL group at any time point (Figure 6). Livers from control animals showed significant lower relative liver weight almost at every time point after the reperfusion in comparison to livers from the BDL group. Significant differences of the relative liver weight between the control and the BDL + T group could only be observed 168 h after reperfusion. 

**Figure 7:**
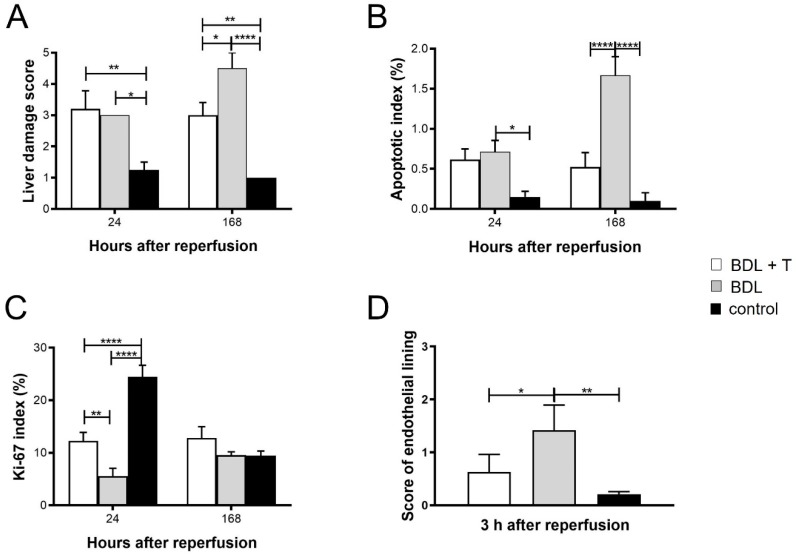
Histopathological changes in livers in the different treatment groups were analyzed in (**A**) Hematoxylin and eosin (HE) staining applying a semi-quantitative score, in (**B**) transferase-mediated d-UTP nick-end labeling (TUNEL) staining shown as apoptotic index (in %), in (**C**) Ki-67 staining measuring proliferation (in %) of Kupffer cells and sinusoidal endothelial cells and infiltration of inflammatory cells and in (**D**) RECA-1 staining in order to score the endothelial lining. Mean and standard deviation are shown in each group with significance levels of * *p* < 0.05, ** *p* < 0.01, **** *p* < 0.0001.

**Figure 8:**
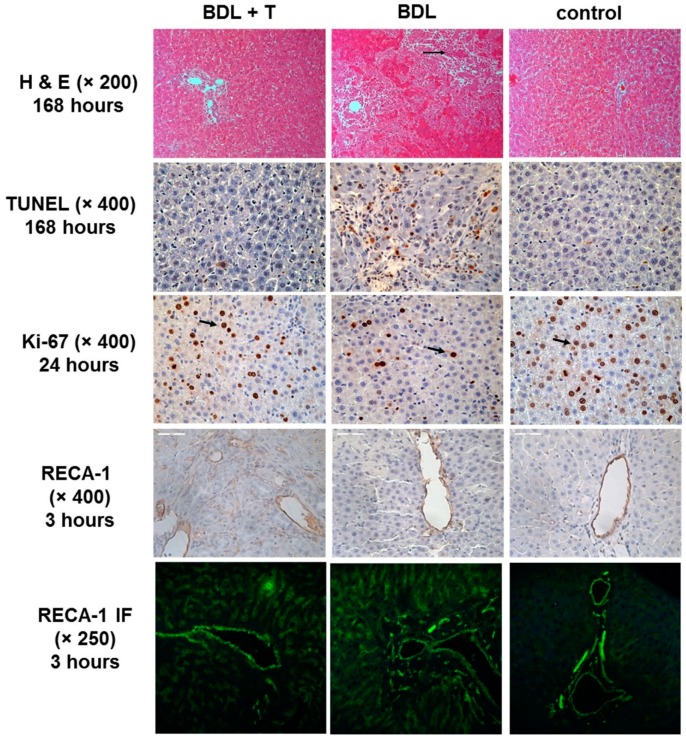
Representative images showing HE staining (1rst row, 200×, 168 h after reperfusion), TUNEL staining (2nd row, 400×, 168 h after reperfusion), Ki-67 stain^in^g (3rd row, 400×, 24 h after reperfusion, arrows), immunohistochemical RECA-1 staining (4th row, 400×), and immunofluorescence RECA-1 staining (5th row, 250×) in liver sections at indicated time points. Arrow in HE section indicates inflammatory cells. Arrows in Ki-67 sections mark proliferating Kupffer cells and sinusoidal endothelial cells.

Further histopathological analysis was performed by semi-quantitative scoring for liver damage analyzing hematoxylin and eosin (HE)-stained liver sections. Apoptotic index was evaluated on transferase-mediated d-UTP nick-end labeling (TUNEL) stained tissue sections. Scores for liver damage and apoptotic index values were comparable between the BDL + T and BDL group at 24 h after reperfusion (Figure 7A,B and Figure 8). However, both scores were significantly lower in the BDL + T group than in the BDL group at 168 h after reperfusion (*p* < 0.05, *p* < 0.0001). The control group showed significant lower liver damage at each time point analyzed compared to BDL or BDL + T group. Significant lower apoptotic index of the control group was only observed in comparison to the BDL group.

With regard to liver regeneration, the Ki-67 labeling index of the BDL + T group was significantly higher than that of the BDL group 24 h after reperfusion (*p* < 0.05), but it was significantly lower compared with the control group (Figure 7C and Figure 8). There was no significant difference among the three groups 168 h after reperfusion. Endothelia lining was visualized via RECA-1 staining and scored. This analysis showed a significant better preserved endothelial lining in the BDL + T group compared to the BDL group (Figure 7D and Figure 8).

Significant histopathological changes were also manifested by the presence of the proliferation of biliary epithelium (Figure 8, Ki-67) with the formation of bile ductules (Figure 8, HE and Figure S2). In addition, proliferation of Kupffer cells and sinusoidal endothelial cells (Figure 8, Ki-67), and infiltration of inflammatory cells were found (Figure 8, HE arrow). These changes were most pronounced in the BDL group and abrogated in the BDL + T group compared to the control group. This was also confirmed by RECA-1 staining for endothelial cells.

Representative staining for HE, TUNEL, Ki-67, and RECA-1 (IHC and 2-Photonmicroscopy) are shown in Figure 8 for the indicated time points and magnifications. More representative pictures of HE stained liver section at a magnification of 100× are shown in Supplemental Figure S2.

## 3. Discussion

We investigated the effect of L-NAME on IRI of cholestatic livers after partial hepatectomy by PM. Therefore, we performed partial hepatectomy of the left lateral lobe and the anterior and posterior caudate lobes (the total resection volume was almost 50%) in cholestatic livers that underwent the PM. The serum AST levels after reperfusion tended to be higher in the BDL + T group (Figure 2A), possibly reflecting increased sinusoidal shear stress due to higher microcirculation in the remnant liver (Figure 2C). It is well accepted that hyperperfusion of the liver results in increased sinusoidal shear stress and leads to small-for-size syndrome in the setting of a reduced-size liver [17,18,19,20,21,22]. Moreover, some studies have reported that a surgical reduction in hepatic blood flow can improve outcome after extended hepatectomy, which indicates that increased sinusoidal shear stress is one of the main causes of liver damage in reduced-size livers [23,24]. Because the eNOS mRNA expression levels were similar between the BDL + T and BDL groups (Figure 5B), improved microcirculation in the remnant liver in the BDL + T group appears to be the result of iNOS inhibition (Figure 5A) [25,26,27]. These results support previous reports that hepatic tissue blood flow was improved by selective iNOS inhibition [28,29]. However, in contrast to our results, previous reports showed lower serum AST levels, possibly reflecting a different mechanism in fibrotic livers.

A relationship between NO levels/concentrations and IRI has been reported [30,31]. NO from eNOS is believed to have cytoprotective effects and to maintain homeostasis [32,33]. The activity of eNOS, however, is significantly decreased in human and rat cirrhotic livers [34,35,36]. Decreased hepatic NO production, especially through reduced activity of eNOS, is considered one of the causes of hepatic IRI in a previous report of warm IRI and liver transplantation models [26,37,38,39]. Most of these studies have shown that a blockade of NO production leads to aggravated liver damage. On the other hand, a large amount of NO production from iNOS has potentially cytotoxic effects because it can react with superoxide anions to form toxic peroxynitrite [38,40,41], and selective inhibition of iNOS prevents apoptosis in acute inflammation and has a damaging effect in IRI [42,43]. The effect of iNOS inhibition was also supported by our present results, and NO in a well-defined concentration range is considered to be important as a trigger for liver regeneration [44,45] and to act as a potent cytoprotective agent [42]. We found that inhibiting the production of NO by L-NAME resulted in significantly decreased liver damage in cirrhotic rats, as shown by histology and improved hepatic microcirculation and regeneration. The beneficial effects in the BDL + T group are thought to result from iNOS inhibition, as higher levels of NOx and iNOS were observed in association with more severe liver damage in the BDL group without L-NAME treatment. Although finding iNOS inhibition after L-NAME treatment is in line with a recent study [26], and the effect of blocking NO to reduce oxidative stress, also in our study (Figure 5C), has been previously reported [46].

In hepatic fibrogenesis, Kupffer cells and hepatic stellate cells (HSCs) are both sources and targets of pro-inflammatory mediators [47,48]. Kupffer cells are the resident hepatic macrophages and an important source of TGF-β in the liver; they also promote HSC activation and fibrogenesis [49,50,51]. HSCs are the main producers of extracellular matrix (ECM) in the injured liver [52]. They reside in the peri-sinusoidal space, and upon liver damage they become activated and transdifferentiate into myofibroblast-like cells in a TGF-β-dependent manner. These cells migrate and accumulate at the sites of tissue repair, secreting large amounts of ECM proteins [53,54]. In this scenario, TGF-β1 appears to be the key mediator of fibrogenesis [55]. The TGF-β1-activated Smad signaling pathway stimulates hepatic fibrosis and is a potential target for therapy [56,57]. In line with that assumption, strategies aimed at disrupting TGF-β1 synthesis and/or signaling pathways markedly decreased fibrosis in several experimental models [58,59]. In addition, HA is considered a biomarker for high-score fibrosis and cirrhosis in various liver diseases. There are some reports indicating that serum HA is useful to diagnose liver damage after hepatectomy and transplantation. It was reported that the serum HA level increases after liver resection [60], suggesting HA as a biomarker to diagnose the rapid progression of fibrosis after liver transplantation [61]. In addition, Valva and colleagues [62], reported that the diagnostic combination of serum levels of HA, TGF-β, and pro-collagen III N-terminal pro-peptide (PIIINP) is more reliable to evaluate the degree of liver fibrosis compared with the serum levels of each marker alone. In support of this notion, we found elevated quantities of TGF-β mRNA, while HA was notably reduced by L-NAME treatment, suggesting that L-NAME interferes with and reduces fibrogenesis after reperfusion (Figure 3B,D and Figure 4B).

Hypoxia-inducible factor (HIF) is one of the principal regulators of the cellular transcriptional responses to hypoxia [63]. This transcription factor plays an important role in triggering cellular protection and metabolic alterations from the consequences of oxygen deprivation. Therefore, HIF activation should be protective against IRI. On the other hand, HIF also contributes to important features of tissue fibrosis and epithelial–to–mesenchymal transition (EMT) [64]. The EMT process has been implicated in the development of tissue fibrosis, and recent studies suggest that HIF-1 plays a role in this process [65,66]. In the mouse BDL model, mice deficient in HIF-1α developed less fibrosis and had fewer activated fibroblasts, suggesting that HIF-1α is an important driving force for the development of liver fibrosis during chronic injury [67]. Furthermore, it has been demonstrated that HIF-1 contributes to EMT by impacting TGF-β signaling through the up-regulation of TGF-β mRNA and protein and through increased TGF-β activity [68,69]. In hypoxic hepatocytes, there is an interaction between HIFs and TGF-β signaling, and TGF-β is considered the key cytokine inducer of EMT [70]. HIF in hepatocytes facilitates TGF-β signaling, and TGF-β signaling is downstream of HIF activation, which leads to hepatocyte EMT [71,72]. Here, we showed a significant increase in HIF-1α after 24 h (Figure 4E) of reperfusion by the addition of L-NAME. The BDL group possessed higher HIF-1α and TGF-β mRNA expression after 168 h of reperfusion. These results confirm the protective effect of L-NAME in reducing IRI by avoiding an increase in TGF-β mRNA expression as evidenced by histopathology and better preserved endothelial lining visualized in RECA-1 immuno-histochemical staining results (Figure 7D and Figure 8).

ADMA is an endogenous inhibitor of NOS [73]. ADMA is synthesized through the enzymatic methylation of L-arginine residues in proteins and is released during proteolysis. Because ADMA is primarily metabolized to citrulline and dimethylamine by the liver enzyme dimethylarginine dimethylaminohydrolase [74], it has been suggested that impaired liver function results in increased plasma levels [74,75] and liver tissue levels of ADMA [76]. Ferrigno et al., have demonstrated the increase of ADMA levels in a BDL model in rats [76]. In contrast ADMA tissues levels were decreased after hepatic ischemia–reperfusion as described by Ferrigno et al. [77].

NOS activity decreases due to dysfunction of eNOS inhibited by ADMA in cirrhotic livers induced by BDL [78]. Moreover, the present study demonstrated that L-NAME prevented the increase of ADMA in the early phase (1 h) after reperfusion (Figure 4C), which correlates with serum AST levels (Figure 2A) and suggests a protective effect of L-NAME against IRI.

## 4. Limitations of the Study

We must admit that the study as presented has several limitations. In our study, we used 1.5 mg/kg body weight of L-NAME that was intravenously injected 15 min before the surgery. Although this dosage produced a restorative effect on the outcome after BDL as assessed by lowered cellular DNA damage (Figure 7), higher Ki-67 labeling index (Figure 7), reduced quantities of NOx (Figure 4A) and iNOS (Figure 5A), decreased levels of TGF-β (Figure 3B,D), and better score of endothelial lining (Figure 7D), we have not systematically optimized the timing and dosing of L-NAME that is most efficient in decreasing oxidative stress in the liver. In this regard, it should also be critically mentioned that pharmacokinetics of L-NAME will be different in humans, which will lead to variation in efficacy, toxicity, and potential side effects that were not analyzed here. Particularly, L-NAME requires hydrolysis of the methyl ester by cellular esterases to become a fully functional inhibitor. The activities of these enzymes might be totally different in rodents and humans. Similarly, it would be interesting to test if the oral application of L-NAME would lead to the same beneficial effects that we have observed after intravenous application.

Last but not least, all studies that we have done were performed in rats and the relevance of our findings for the human situation was not tested. The model we used in our study relying on BDL combined with a 50% partial hepatectomy with PM is rather artificial. Blood loss during liver resection, hepatic inflow occlusion, and hepatic ischemia–reperfusion injury in patients with cholestatic, fibrotic, or cirrhotic livers might be more complex than mimicked in our protocol. All these limitations will be improved in future studies.

## 5. Materials and Methods

### 5.1. Ethical Approval

The animal protocol was approved by the Governmental Animal Care and Use Committee, which is the Landesamt für Umwelt-, Natur- und Verbraucherschutz (LANUV, North Rhine Westphalia, Recklinghausen, Germany) (project identification code: AZ: 84-02.04.2011.A103). All experiments were performed in accordance with the German legislation governing animal studies following the Guide for the Care and Use of Laboratory Animals (NIH publication, 8th edition, 2011) and the 2010/63/EU Directive on the protection of animals used for scientific purposes (Official Journal of the European Union, 2010).

### 5.2. Animals

Male Wistar rats weighing 200 to 300 g were housed with free access to water and a standard diet (ssniff-Spezial Diäten GmbH, Soest, Germany) under specific pathogen-free conditions according to the guidelines of the Federation of European Laboratory Animal Science Associations (FELASA) in a temperature- and humidity-controlled environment with a 12 h light and dark cycle. The rats were randomly divided into three groups of 16 rats: (i) the control (non-bile duct ligation (BDL)/no treatment), (ii) the BDL + T (BDL/L-NAME treatment), and (iii) the BDL (BDL/no treatment) group.

### 5.3. Surgical Procedure

#### 5.3.1. Induction of Biliary Cirrhosis

Rats in the BDL + T and BDL group underwent BDL surgery to induce biliary cirrhosis 14 days before the IRI experiment. After induction of general anesthesia by inhalation of 5 vol% isoflurane and 5 L/min oxygen (Abbott GmbH & Co. KG, Wiesbaden, Germany) for approximately 2 min, anesthesia was maintained by reducing the isoflurane to 2 vol% with 2 L/min oxygen. Pre-operative prophylactic antibiotic treatment and analgesia were achieved by subcutaneous injection of cefuroxime sodium (g/kg/24 h; Cefuroxim, Fresenius Kabi GmbH, Bad Homburg, Germany) and buprenorphine (0.1 mg/kg/24 h) (Temgesic; EssexPharma, Munich, Germany) until 3 d after surgery. The abdomen was opened by a midline laparotomy. The common bile duct was identified and doubly ligated using 6-0 polydioxanone absorbable monofilament (PDS II; Ethicon, Inc., Somerville, NJ, USA). The abdominal wall was closed in two layers (both running suture for peritoneum and fascia and interrupting suture for skin with 4-0 Vicryl (Ethicon, Norderstedt, Germany)).

#### 5.3.2. Partial Hepatectomy and the IRI Experiment

Fourteen days after bile duct ligation a partial hepatectomy with PM using a Satinsky vascular clamp was performed on all of the rats in each group. The left lateral lobe and the anterior and posterior caudate lobes were ligated with 4-0 silk (Resorba, Nürnberg, Germany) and resected as 50% partial hepatectomy. After partial hepatectomy, inflow occlusion was released by declamping the hepatoduodenal ligament. Finally, all groups were injected intraperitoneally with 5 mL of sterile isotonic saline solution. The rats in the BDL + T group were injected with L-NAME (1.5 mg/kg obtained from Merck KGaA, Darmstadt, Germany) through the penile vein 15 min before PM.

### 5.4. Hepatocellular Damage

Serum levels of aspartate aminotransferase (AST) at 1, 3, 24, and 168 h after reperfusion were measured as described previously [79].

### 5.5. Measurement of Portal Venous Flow (PVF) and Microcirculation of Liver

Portal venous flow (PVF) and microcirculation of the liver were measured before PM and at the time of sacrifice. PVF was evaluated with a transit-time perivascular flowmeter (T403; Transonic Systems, Inc., Ithaca, NY, USA), and microcirculation of the liver was assessed by combined laser Doppler flowmetry and spectrophotometry device (O2C: oxygen to see; LEA Medizintechnik GmbH, Giessen, Germany).

### 5.6. Lipid Peroxidation

To assess the impact of oxygen free radical activity after reperfusion, we measured the malondialdehyde (MDA) concentration as described previously [80]. Briefly, 100 μL of serum was mixed with 750 μL of 0.44 M phosphoric acid, 250 μL of 42 mM aqueous solution of thiobarbituric acid, and 500 μL of water. Samples were incubated in boiling water for 60 min and then immediately cooled on ice. Fluorescent lipid peroxidation/thiobarbituric acid adduct were measured using a fluorescence spectrophotometer (Tecan Infinite, Tecan Deutschland GmbH, Crailsheim, Germany). Different dilutions of tetraethoxypropane were used as the external standard.

### 5.7. Pro-Inflammatory Cytokines

Serum levels of interleukin (IL)-6 and transforming growth factor-β (TGF-β) at 3, 24, and 168 h after reperfusion were measured by rat enzyme-linked immunosorbent assay (ELISA) kits (R&D Systems, Minneapolis, MN, USA) according to the manufacturer’s instructions.

### 5.8. Serum Hypoxia-Inducible Factor-1α (HIF-1α) Levels

Serum levels of HIF-1α at 3, 24, and 168 h after reperfusion were measured using ELISA kits (MyBioSource, San Diego, CA, USA) according to the manufacturer’s instructions.

### 5.9. mRNA Expression Analysis of iNOS, eNOS, IL-6 and TGF-β by Quantitative Real-Time PCR

Reverse-transcription quantitative polymerase chain reaction (RT–qPCR) analyses were performed as described previously [81]. In short, total RNA was isolated from 30 mg of liver tissue samples, and 250 ng of RNA was used for complementary DNA (cDNA) synthesis using a High-Capacity cDNA Reverse Transcription Kit (Applied Biosystems, Carlsbad, CA, USA) according to the manufacturer’s instructions. The following primers and probes from Applied Biosystems were used: glyceraldehyde-3-phosphate dehydrogenase (GAPDH, Rn_01775763_g1); iNOS (Rn_00561646_m1); eNOS (Rn_02132634_s1); IL-6 (Rn_1410330_m1); and TGF-β (Rn_00572010_m1). Relative mRNA expression of iNOS, eNOS, IL-6, and TGF-β were normalized to GAPDH expression and shown in comparison to the control group (baseline level 1 in the graph).

### 5.10. NOx, Hyaluronic Acid (HA) and Asymmetric dimethylarginine (ADMA) in Liver Tissue

NOx, HA, and ADMA in liver tissue at 1 h and 24 h after reperfusion was measured by the Griess reaction assay using ELISA kits (NO: Cayman Chemical Company, Ann Arbor, MI, USA; HA: TECO medical AG, Sissach, Switzerland; ADMA: Immundiagnostik AG, Bensheim, Germany) according to the manufacturer’s instructions.

### 5.11. SDS-PAGE and Western Blot Analysis

Liver protein extracts were prepared in RIPA buffer containing 20 mM Tris-HCl (pH 7.2), 16 mM NaCl, 2% (w/v) NP-40, 0.1% (w/v) SDS, 0.5% (w/v) sodium deoxycholate and the Complete™-mixture of proteinase inhibitors (Merck KGaA, Darmstadt, Germany). Equal amounts of tissue protein extracts (40 µg) were diluted with Nu-PAGE™ LDS electrophoresis sample buffer with DTT as a reducing agent, heated at 95 °C for 10 min, and separated in 4–12% Bis-Tris gradient gels, using MOPS or MES running buffers (all from Invitrogen). Proteins were electro-blotted onto nitrocellulose membranes (Schleicher & Schuell BioScience, Dassel, Germany), and equal loading was shown in Ponceau S stain. Subsequently, non-specific binding sites were blocked in TBS containing 5% (w/v) non-fat milk powder. For immune-detection of NOS2 (iNOS) or NOS3 (eNOS), the antibodies (sc-649, sc-654) were diluted in 2.5% (w/v) non-fat milk powder in Tris-buffered saline and incubated overnight at 4 °C. The primary antibodies were visualized using horseradish peroxidase conjugated anti-rabbit IgG (Santa Cruz Biotech, Santa Cruz, CA, USA) and the SuperSignal chemiluminescent substrate (Pierce, Bonn, Germany).

Densitometry of Western blots was done using the open source image processing program ImageJ software, version 1.46r [82]. For relative quantification of NOS3 (eNOS), we used the upper protein band detected in our Western blot analysis.

### 5.12. Histopathology

Liver tissue samples were collected at the time of sacrifice and relative liver weights were calculated. For light microscopy, sections were fixed in 4% neutral buffered formalin and embedded in paraffin. The specimens were sectioned into 2–4 μm thick slices using a microtome and stained with hematoxylin and eosin (HE). Injury in each specimen was graded to the extent of the region in five randomly chosen, non-overlapping fields (400× magnification) using a Leica DM 2500 microscope (Leica, Wetzlar, Germany). Lesions were graded on a scale from 1 to 4: 1, no changes or negligible lesions affecting 0–10% of the field; 2, mild lesions affecting 10–40% of the field; 3, moderate lesions affecting 40–70% of the field; and 4, severe lesions affecting >70% of the field. All tissue sections were examined in a blinded fashion by two independent investigators including a board-certified pathologist (M.A.).

### 5.13. TUNEL-Staining for Apoptosis Measurement

Apoptosis was analyzed by terminal deoxynucleotidyl transferase-mediated d-UTP nick-end labeling (TUNEL) staining using digoxigenin-labeled UTP detected with an antibody to digoxigenin using an in-situ apoptosis detection kit (Apoptag; CHEMICON, Schwalbach am Taunus, Germany), as described previously [83]. The apoptotic index also represented the percentage of TUNEL-positive cells relative to the total number of hepatocytes (400× magnification, five fields in each rat).

### 5.14. Immunohistochemistry (IHC) for Ki-67 and RECA-1

To assess the cell proliferation rate, tissue sections were subjected to immunohistochemical testing using monoclonal mouse Ki-67 (MIB5, 1:10; DAKO, Glostrup, Denmark). The Ki-67 labeling index represented the percentage of hepatocytes with Ki-67-positive nuclei relative to the total number of hepatocytes (400× magnification, five fields for each rat). For the analysis RECA-1 expression in the liver, the tissue sections were subjected to antigen retrieval by heating the sections for 24 min using a steamer in 0.1 mol/L of citrate buffer (pH 6.0) followed by slow cool down cycle to room temperature. After the blocking of nonspecific binding sites with 5% normal rabbit serum in a 2% skim milk solution in phosphate buffered saline (PBS) for 60 min, the sections were incubated overnight at 4 °C with a mouse monoclonal antibody against rat RECA-1 (10 ug/mL, abcam, Cambridge, UK, ab9774) at 4 °C followed by incubation with a 1:300 dilution of a biotin-labeled rabbit anti-mouse secondary antibody (DAKO, Hamburg, Germany E0413) for 30 min at room temperature. Finally, the slides were incubated with the DAB substrate (Merck KGaA, Darmstadt, Germany, D5905) for 5 min before undergoing Mayer’s hematoxylin counterstaining for 60 s and being mounted. Endothelial lining was analyzed based on the RECA-1 staining, which specifically stains vascular endothelium. On a scale from 0 to 3: 0, no changes, intact thin endothelium layer, no interruptions of the endothelium layer; 1, mild changes; 2, moderate changes; and 3, severe changes based on thickening of the endothelium layer and interruptions of the endothelium. For the analysis 6 different vessels per liver sections were analyzed and mean and standard deviations were calculated.

### 5.15. Immunofluorescence (IF) Detection of RECA-1

For immunofluorescence analysis liver tissues were shock frozen in liquid nitrogen and stored at −80 °C. Liver tissues were embedded in Tissue-Tek^®^ O.C.T. Compound (Sakura^®^ Finetek, Netherland) and 2–4 μm tissue sections were prepared with a cryo-microtome. After fixation of the tissue sections for 10 min with acetone, nonspecific binding sites were blocked by incubation with a 10% normal rabbit serum in PBS for 20 min followed by an 1 h incubation at room temperature with the mouse monoclonal antibody against rat RECA-1 (10 µg/mL, abcam, Cambridge, UK, ab9774). Tissue sections were then incubated with an Alexa Fluor 488 coupled goat anti-mouse IgG antibody for 1 h in the dark (1:500, abcam, ab150117) and finally mounted with Roti^®^-Mount FluorCare (Carl Roth, Karlsruhe, Germany) and analyzed by a FluoView1000MPE Two-Photon Laser Scanning Microscopy (Olympus Corp., Tokyo, Japan) equipped with a 25× NA1.05 water dipping objective. For excitation of Alexa Fluor 488 a Ti:Sapphire laser (MaiTai DeepSee, Spectra Physics, Mountain View, USA) was tuned to the wavelength of 800 nm. The emission of Alexa Fluor 488 was collected at 495–540 nm. The autofluorescence originating from the tissue was detected in two additional emission ranges of 419–465 nm and 590–630 nm. Series of subsequent xy-frames with 1024 × 1024 pixels in 1 µm z-steps were obtained for visualization of the complete tissue slide. Images shown as z-projection of the image stacks used the software FluoView ASW (Olympus GmbH, Hamburg, Germany) and merged to the detection channels.

### 5.16. Statistical Analyses

The data are expressed as the mean ± standard error of the mean. One-way analysis of variance (ANOVA) was used to test the differences among the three groups. Two-way analysis of variance (ANOVA) with the Bonferroni or Tukey post-test was used to assess time-dependent changes and differences between groups at each time point. A *p*-value of <0.05 was considered significant. Analyses were performed using GraphPad Prism 8 Software (GraphPad Software Inc., San Diego, CA, USA).

## 6. Conclusions

In conclusion, we demonstrated that L-NAME improved microcirculation and regeneration of the cholestatic liver after partial hepatectomy by PM. These effects of L-NAME may be due to the prevention of iNOS and TGF-β up-regulation.

## Supplementary Materials

The supplementary materials are available online at https://www.mdpi.com/1422-0067/20/9/2114/s1.

## Abbreviations

ADMA	Asymmetric dimethylarginine
AST	aspartate aminotransferase
BDL	Bile duct ligation
BDL + T	Bile duct ligation + Treatment (L-NAME)
cNOS	constitutively expressed NOS
DAB	3,3-Diaminobenzidine
ECM	Extracellular matrix
EMT	Epithelial-to-mesenchymal transition
eNOS	Endothelial NOS
HA	Hyaluronic acid
HIF-1α	Hypoxia-inducible factor-1α
HSC	Hepatic stellate cells
IF	Immunofluoresence
IHC	Immunohistochemistry
IL-6	Interleukin 6
iNOS	Inducible NOS
IRI	Ischemia–reperfusion injury
L-NAME	N-Nitroarginine methyl ester
MDA	Malondialdehyde
mRNA	Messenger ribonucleic acid
nNOS	Neuronal NOS
NO	Nitric oxide
NOS	NO synthase(s)
PM	Pringle maneuver
PVF	Portal venous flow
RT-qPCR	Quantitative real-time polymerase chain reaction
RECA-1	Rat endothelial cell antigen-1
TGF-β	Transforming growth factor β
