# Supplementary material for: The Impact of a Nitric Oxide Synthase Inhibitor (L-NAME) on Ischemia–Reperfusion Injury of Cholestatic Livers by Pringle Maneuver and Liver Resection after Bile Duct Ligation in Rats
Source: Int J Mol Sci. 2019 Apr 29;20(9):2114. doi: 10.3390/ijms20092114 (PMC6539833; doi:10.3390/ijms20092114)
Supplement: Supplementary file 1 [file ijms-20-02114-s001.pdf]

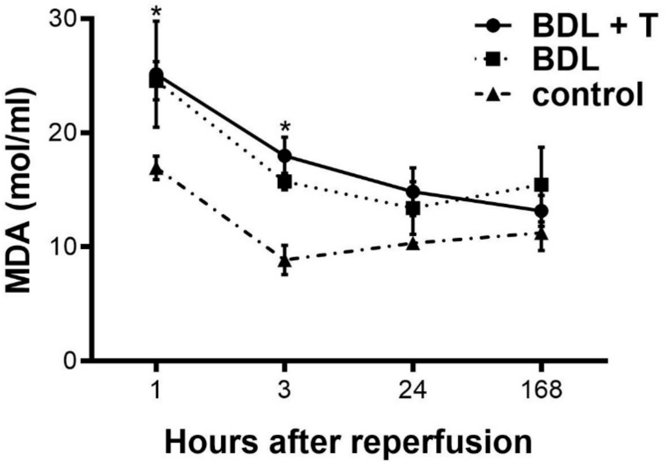

Supplemental Figure 1. To estimate the oxygen free radical activity in the liver, lipid peroxidation in serum was determined by malondialdehyde (MDA) measurements at 1, 3, 24, and 168 h after reperfusion. Mean and standard deviation are shown in each group. Mean and standard deviation are shown in each group with significance levels of $^*p < 0.05$.

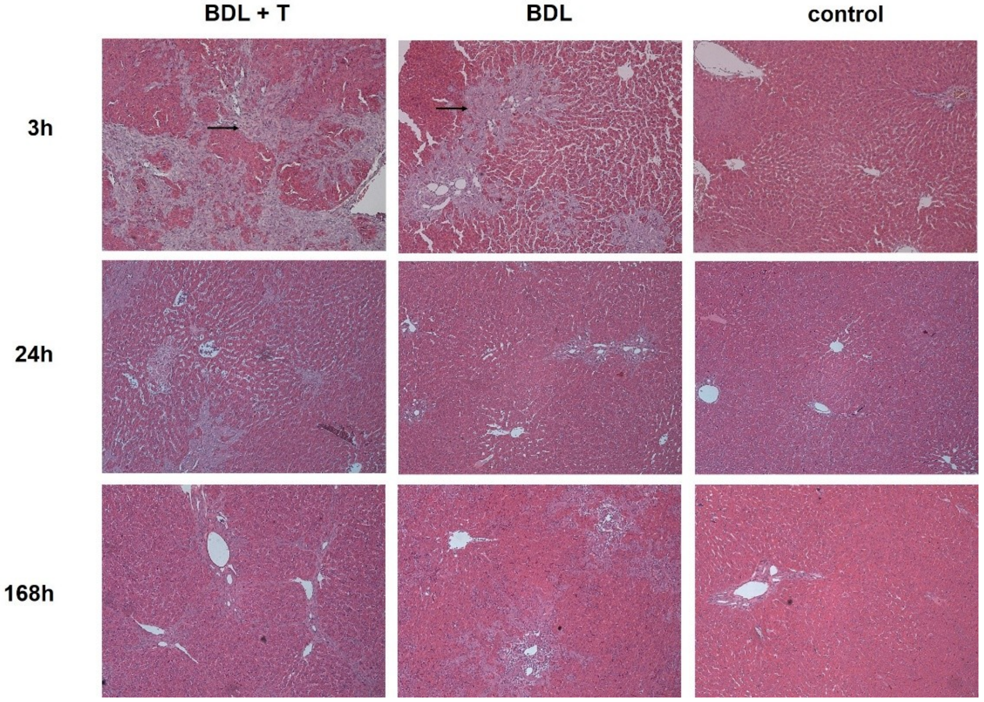

Supplemental Figure 2. Representative images showing HE stained liver sections at indicated time points and the treatment groups BDL + T, BDL,s and control (100x). Arrows mark fibrous connective tissue.
